# Higher-Dimensional Fractional Order Modelling for Plasma Particles with Partial Slip Boundaries: A Numerical Study

**DOI:** 10.3390/nano11112884

**Published:** 2021-10-28

**Authors:** Tamour Zubair, Muhammad Imran Asjad, Muhammad Usman, Jan Awrejcewicz

**Affiliations:** 1 School of Mathematical Sciences, Peking University, Beijing 100871, China; tamourzubair@pku.edu.cn; 2 Department of Mathematics, University of Management and Technology, Lahore 40050, Pakistan; Imran.asjad@umt.edu.pk; 3 Department of Mathematics, National University of Modern Languages (NUML), Islamabad 44000, Pakistan; dr.usman@numl.edu.pk; 4 Department of Automation, Biomechanics, and Mechatronics, Faculty of Mechanical Engineering, Lodz University of Technology, 90-924 Lodz, Poland

**Keywords:** partial slip boundary conditions, polynomial theory, linear polarization, fraction plasma modelling

## Abstract

We integrate fractional calculus and plasma modelling concepts with specific geometry in this article, and further formulate a higher dimensional time-fractional Vlasov Maxwell system. Additionally, we develop a quick, efficient, robust, and accurate numerical approach for temporal variables and filtered Gegenbauer polynomials based on finite difference and spectral approximations, respectively. To analyze the numerical findings, two types of boundary conditions are used: Dirichlet and partial slip. Particular methodology is used to demonstrate the proposed scheme’s numerical convergence. A detailed analysis of the proposed model with plotted figures is also included in the paper.

## 1. Introduction

In the study of plasma [1,2,3,4,5,6,7] particles, there is a ground breaking tool available in the literature named the “Vlasov Maxwell system”, which is the amalgamation of the Vlasov equation and Maxwell equations. The Vlasov and Maxwell equations (MEs) are actually the mathematical formulation of performance of plasma particles and electro-magnetic field, formulated by well-known scientists Anatoly Vlasov (1938) and James Clerk Maxwell (1862), respectively. This system provides us different types of information in three-dimensional velocity **v** and position **r** coordinates under the impacts of electromagnetism. The mathematical formulation of this system is given below [8,9,10]:

(1) $$\begin{array}{c} D_{t}^{\alpha }f+v\cdot \nabla_{r}f+\frac{q}{m}\underset{*}{\underbrace{\left(E+v\times B\right)}}\cdot \nabla_{v}f=0,\\ {}*\{\begin{array}{c} \frac{1}{c^{2}}D_{t}^{\alpha }E-\nabla \times B+\mu_{0}J=0,D_{t}^{\alpha }B+\nabla \times E=0,\\ \nabla \cdot E=\frac{n}{\varepsilon_{0}},\nabla \cdot B=0, \end{array} \end{array}\}\;$$

**Figure 1:**
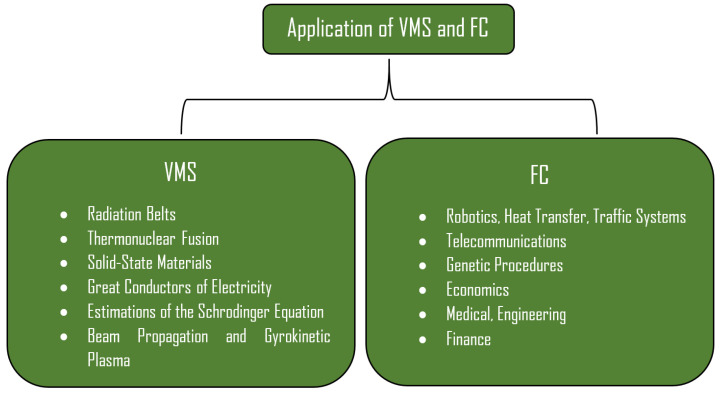
Applications of VMS and FC.

In the above system defined in Equation (1), *f* represents distribution function and also $f=f\left(t,r,v\right),r,v\in {\mathbb{R}}^{ϒ},ϒ=1,2,3$. ME’s are included due to (*) part of system (1). The importance of VMS is also due to the frequent applications which are given in [9,11,12,13]. $D_{t}^{\alpha }$ is the time-fractional operator [8], which can be seen in the system (1) to belong to the fractional calculus (FC). Fractional calculus is another innovative idea to explore the concealed procedures of the physical nature. With the help of literature study, it can be easily noticed that fractional calculus was the topic of pure-mathematics, but in a very short time, this theory has become famous due to the large number of applications [14,15,16,17,18,19]. FC turns the directions of research and generates new ideas to study the existing models in a different manner. The applications of VMS and FC are explained in Figure 1.

**Figure 2:**
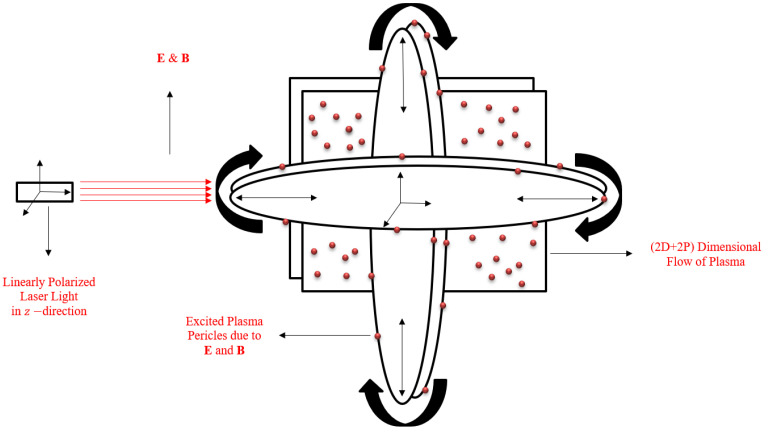
Geometry of the problem.

The first goal of this study is to transform the defined physical geometry into a mathematical model. For this purpose, we defined our geometry in Figure 2. The simple description of this geometry is in the following points as [8,20,21]:

- Collisionless plasma particles are available in a higher-dimensional computational domain, and LASER light is settled in such a way that it creates light with linear polarization properties. Particles are actually charged particles, and they generate a self-consistent electro-magnetic field.
- As light generates electromagnetism, it has its own fields in the computational domain.
- According to the geometry, we have the following components of electric **E**, vector potential **A** and magnetic **B** are given:

$$A=A\left(t,x,y\right)=0,0,A_{z}\hat{k},B=B\left(t,x,y\right)=B_{x}\hat{i}+B_{y}\hat{j},E=E\left(t,x,y\right)=E_{x}\hat{i}+E_{y}\hat{j}+E_{z}\hat{k},$$

According to the assumptions, we consider the distribution function as:

(2) $$f(t,r,P)=f(t,x,y,p_{x},p_{y})\delta (p_{z}-p_{0}(t,x,y)),\delta :\mathbb{R}\times \mathbb{R}\to \mathbb{R},$$

In above Equation (2), $p_{0}\left(t,x,y\right)$ and δ are the momentum component and “Dirac measure”, respectively. Further, we use the concepts of Hamiltonian, Coulomb gauge and canonical conjugate momentum to obtain the dimensionless form of the system as [9,20,21]:

(3) $$\begin{array}{c} f^{\alpha }+\frac{p_{x}}{\gamma_{1}}\frac{\partial f}{\partial x}+\frac{p_{y}}{\gamma_{1}}\frac{\partial f}{\partial y}-\left(E_{x}+\frac{A_{z}}{\gamma_{2}}\frac{\partial A_{z}}{\partial x}\right)\frac{\partial f}{\partial p_{x}}-\left(E_{y}+\frac{A_{z}}{\gamma_{2}}\frac{\partial A_{z}}{\partial y}\right)\frac{\partial f}{\partial p_{y}}=0,\\ A_{z}^{2\alpha }-\left(\frac{\partial^{2}}{\partial x^{2}}+\frac{\partial^{2}}{\partial y^{2}}\right)A_{z}+n_{\gamma }A_{z}=0,\\ E_{x}^{\alpha }-j_{x}=0,\\ \begin{array}{c} E_{y}^{\alpha }-j_{y}=0,\\ \begin{array}{c} n_{\gamma }=\int_{\mathbb{R}}^{}\frac{1}{\gamma_{2}}fdp_{x}dp_{y},j_{x}=\int_{\mathbb{R}}^{}\frac{p_{x}}{\gamma_{1}}fdp_{x}dp_{y},\\ j_{y}=\int_{\mathbb{R}}^{}\frac{p_{y}}{\gamma_{1}}fdp_{x}dp_{y},n=\int_{\mathbb{R}}^{}fdp_{x}dp_{y}. \end{array} \end{array} \end{array}\}$$

The suitable generalized partial slip boundary conditions for Equation (3), are given below [22,23,24];

$$\begin{array}{c} {\left(f+\Lambda_{f}\frac{\partial f}{\partial x}\right)|}_{x=0,L_{x}}=0,{\left(f+\Lambda_{f}\frac{\partial f}{\partial y}\right)|}_{y=0,L_{y}}=0,{\left(f+\Lambda_{f}\frac{\partial f}{\partial p_{x}}\right)|}_{p_{x}=0,L_{p_{x}}}=0,{\left(f+\Lambda_{f}\frac{\partial f}{\partial p_{y}}\right)|}_{p_{y}=0,L_{p_{y}}}=0,\\ \begin{array}{c} {\left(A_{z}+\Lambda_{A_{z}}\frac{\partial A_{z}}{\partial x}\right)|}_{x=0,L_{x}}=0,{\left(A_{z}+\Lambda_{A_{z}}\frac{\partial A_{z}}{\partial y}\right)|}_{y=0,L_{y}}=0,{\left(A_{z}+\Lambda_{A_{z}}\frac{\partial A_{z}}{\partial p_{x}}\right)|}_{p_{x}=0,L_{p_{x}}}=0,{\left(A_{z}+\Lambda_{A_{z}}\frac{\partial A_{z}}{\partial p_{y}}\right)|}_{p_{y}=0,L_{p_{y}}}=0,\\ \begin{array}{c} {\left(E_{x}+\Lambda_{E_{x}}\frac{\partial E_{x}}{\partial x}\right)|}_{x=0,L_{x}}=0,{\left(E_{x}+\Lambda_{E_{x}}\frac{\partial E_{x}}{\partial y}\right)|}_{y=0,L_{y}}=0,{\left(E_{x}+\Lambda_{E_{x}}\frac{\partial E_{x}}{\partial p_{x}}\right)|}_{p_{x}=0,L_{p_{x}}}=0,{\left(E_{x}+\Lambda_{E_{x}}\frac{\partial E_{x}}{\partial p_{y}}\right)|}_{p_{y}=0,L_{p_{y}}}=0,\\ {\left(E_{y}+\Lambda_{E_{y}}\frac{\partial E_{y}}{\partial x}\right)|}_{x=0,L_{x}}=0,{\left(E_{y}+\Lambda_{E_{y}}\frac{\partial E_{y}}{\partial y}\right)|}_{y=0,L_{y}}=0,{\left(E_{y}+\Lambda_{E_{y}}\frac{\partial E_{y}}{\partial p_{x}}\right)|}_{p_{x}=0,L_{p_{x}}}=0,{\left(E_{y}+\Lambda_{E_{y}}\frac{\partial E_{y}}{\partial p_{y}}\right)|}_{p_{y}=0,L_{p_{y}}}=0. \end{array} \end{array} \end{array}\}$$

The above relations change the boundaries into

- Partial slip boundary conditions (SPSBCs) using $\Lambda_{f}=\Lambda_{A_{z}}=1,\Lambda_{E_{x}}=\Lambda_{E_{y}}=0$.

- Dirichlet boundary conditions (DBCs) using $\Lambda_{f}=\Lambda_{A_{z}}=\Lambda_{E_{x}}=\Lambda_{E_{y}}=0$.

The most frequently and broadly used methods for VMS are particle-in-method (PIM) [12,25,26]. Grid-dependent methods such as finite volume [27], finite element [28,29,30,31,32,33] and finite difference [11] and Galerkin [28,34,35] methods can also be inspected in the literature for VMS. D. Nunn [36] suggested an algorithm, which is a combination of spline and Fourier concepts. As we have mentioned that our modelled problem is a fractional order VMS system, so we can say it is a system of fractional differential equations (FDEs). FDEs are also treated numerically, which can be found in the literature. Some recent concepts of VMS, FDEs and the numerical simulations can be deliberate from the refs. [8,27,37,38] and [39,40,41,42], respectively. T. Zubair reported in ref. [8] that the scheme is highly efficient and holds all the points discussed about numerical strategy in the start of this subsection. Therefore, we are going to extend this scheme to the higher dimensional problem along with partial-slip effects of boundaries in this paper. For this determination; we modify the Gegenbauer polynomials. Therefore, we are bounded to select or articulate the numerical strategy that covers all the above points. The idea to study VMS and FC collectively for higher dimensions is not reported yet in the literature except the ref. [8], in which the author of this current paper itself discussed the model, but it is a lower dimensional model.

The main idea of this paper is that we formulated an extended version of VMS using the concepts of fractional calculus and further numerical simulations of the problem, which is influenced by the concepts of partial slip boundaries [22,23,24], with the help of a modified algorithm. For this purpose, we strategized a specific geometry with partial slip boundaries and further verbalized the assumptions using the basic perceptions and theorems available in the literature [9,37,43,44]. We suggested the suitable modified version of a numerical algorithm. The numerical outcomes are validated using different approaches explained in the proceeding section of the paper. All the ideas discussed above, i.e., fractional concepts, modified numerical algorithm and partial slip, incorporated on Vlasov Maxwell system, which open new ways to study the problem which is not focused by researchers yet.

The presented study is separated into different sections. The first section consists of a detailed literature survey, creation of a problem, and applications. In the second portion, we have provided the knowledge regarding the modified numerical system. The third component consists of extensive investigation of the numerical outcome. In the last section, we concluded our offered study. This study developed new standards that can further motivate the readers to extend it to the Boltzmann approximations.

## 2. Formulation of Numerical Scheme

**Figure 3:**
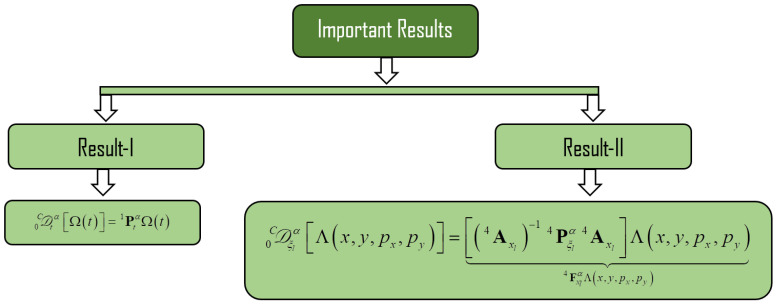
Important results.

The function approximations are [8,20,21]:

f˜(x,y,px,py)=∑i=1M1∑j=1M2∑k=1M3∑l=1M4ρi,j,k,lGi,j,k,lμ(x,y,px,py)=K4TΛ(x,y,px,py),

and also:

K4=[ρ1, ρ2,ρ3,…,ρr]T,Λ=[G1μ, G2μ,G3μ,…,Grμ]T,r=M4(M3(M2(i1−1)+j1−1)+k1−1)+l1,

where the vectors K4 and Λ are of $M_{1}M_{2}M_{3}M_{4}\times 1$ order. Some of the important results are listed below (see Figure 3) as:

In result-I, Caputo fractional differentiation with order γ−1<α<γ, $\alpha \in {\mathbb{R}}^{+}$ is defined, and P1tα is square matrix with order M×M can written as [20,21,44]:

P1tα=t−α[0⋯00⋯00⋮⋱⋮⋮⋯⋮⋮0⋯γ!Γ(γ−α+1)0⋯0⋮0⋯0(γ+1)!Γ(γ−α+2)⋯0⋮⋮⋯⋮⋮⋱⋮⋮0⋯⋮⋮⋯(M−2)!Γ(M−α−1)00⋯⋮⋮⋯0(M−1)!Γ(M−α)]

The definition of Caputo fractional derivative is

D0Ctαςj=j!Γ(j−α+1)tj−α,  j=⌈ρ⌉,⌈ρ⌉+1,…,M−1,

Here, ρ−1<α<ρ and $\alpha \in {\mathbb{R}}^{+}$.

As we have the four-dimensional function, therefore the fractional order matrix form of differentiation is given in result-II. According to the variable we have,

F4x1α=F4xα, F4x2α=F4yα, F4x3α=F4pxα,F4x4α=F4pyα,A4x1=A4x,P4x1α=P4xα,F4x1α=F4xα, F4x2α=F4yα, F4x3α=F4pxα,F4x4α=F4pyα,A4x1=A4x,P4x1α=P4xα,}

**Figure 4:**
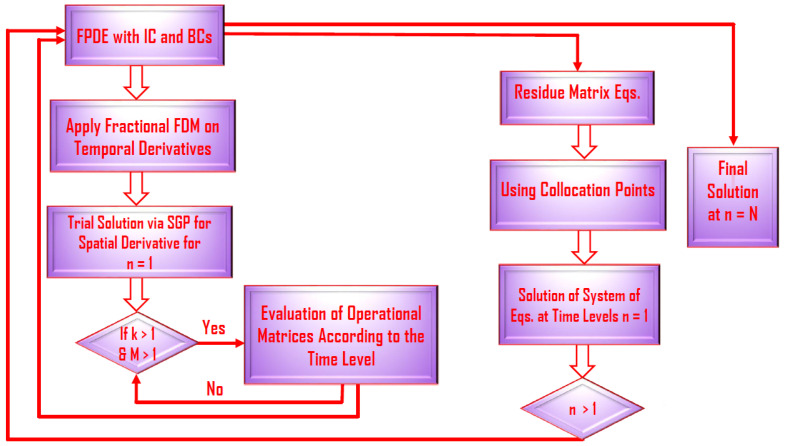
Flow chart of the methodology.

The above defined square matrices are of order $M_{1}M_{2}M_{3}M_{4}\times M_{1}M_{2}M_{3}M_{4}$ and are explained in detail in refs. [8,20,21]. Finally, on the bases of results and ref. [8], methodology is defined in a flow chart that can be seen in Figure 4.

The above methodology will be initiated with the help of the following initial data as follows [45,46]:

$$f\left(0,x,y,p_{x},p_{x}\right)=\frac{1}{\sqrt{2\pi }}e^{\frac{-\left(p_{x}^{2}+p_{y}^{2}\right)}{2}}\left(1+\varepsilon_{1}\cos\left(k_{x}x\right)+\varepsilon_{2}\cos\left(k_{x}y\right)\right),$$

The initial condition described above is referred to as a “2D Maxwellian two-stream cosine perturbation”, and we consider $k_{x}=k_{y}=0.5$ and $\varepsilon_{1}=0.25,\varepsilon_{2}=0.35$. This particular initial condition settled for the data is to boost up the complexities of the problem, so that with this complex initial perturbation, the presented scheme has been tested. The generic Python and MAPLE 13 codes have been developed, and several simulations have been run to obtain the necessary results. To demonstrate the numerical structure’s competency, we presented and discussed the numerical convergence and stability of the projected technique using a distinct approach.

## 3. Discussion about Numerical Results

As we know that convergence and stability of the numerical strategy are very important, we are also aware that the exact and close form solution is not available in the literature yet. With this prospective, we choose two different methods to validate the numerical convergence of the formulated algorithm. The complete detail of both methods is given in ref. [8,20,21]. In this method, we use the concepts of norm of two consecutive iterations, which can be seen in ref. [8] in detail. There are two types of numerical values such as integer and fractional values of fractional parameter is used.

**Figure 5:**
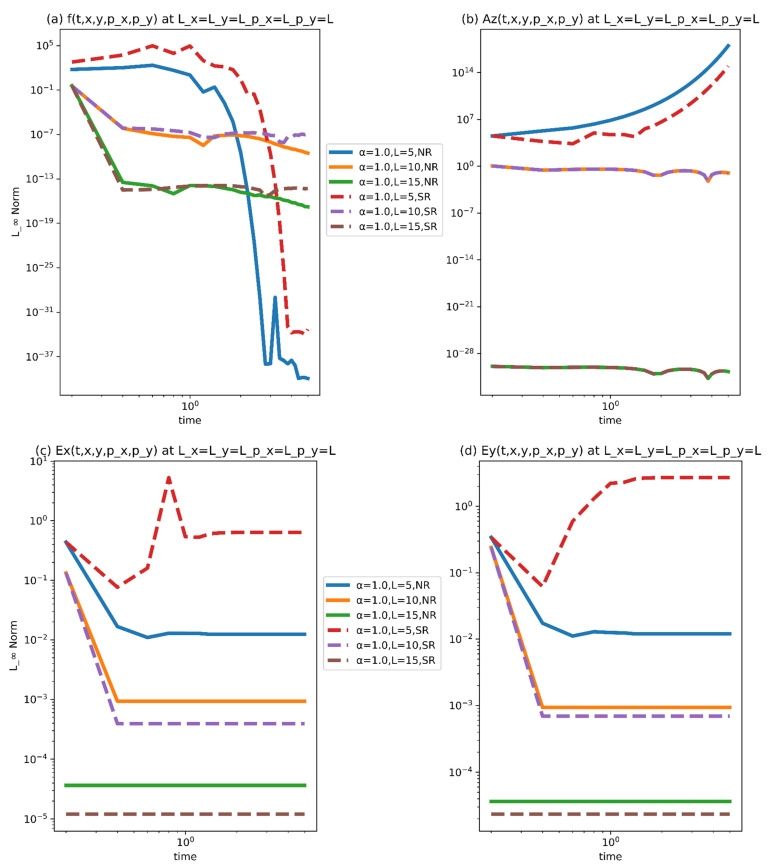
(**a**–**d**) Norm plots for the NR and SR case at $M_{1}=M_{2}=M_{3}=M_{4}=5,N=1000,\alpha =1.0$.

**Figure 6:**
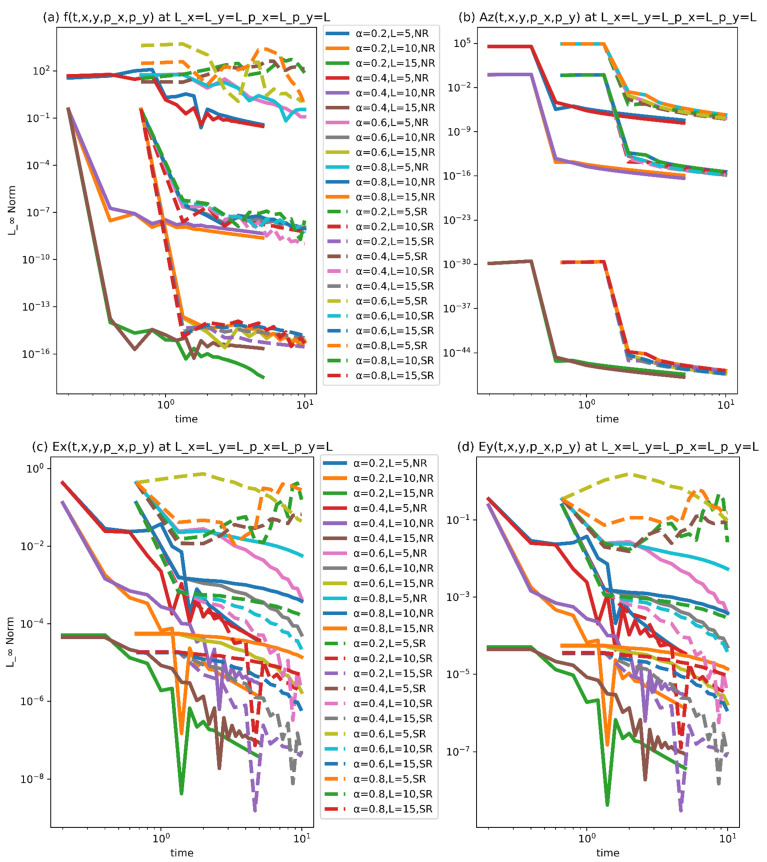
(**a**–**d**) Convergence of norm at $N=1000,M_{1}=5,M_{2}=M_{3}=M_{4}=M_{1}$.

We can simply conclude from Figure 5 and Figure 6 that the proposed algorithm is accurate and compatible with this model. The pivotal factor is that the technique is stable and numerical convergence increases as the computing domain increases for both NR and SR scenarios. In light of the intricacies of the problem stated in the preceding part, we can simply conclude that the recommended method is capable of covering the problem’s physics better. In our current study, we have the following parameters.

- NR and SR
- DBCs and PSBCs
- Fractional parameter α

**Figure 7:**
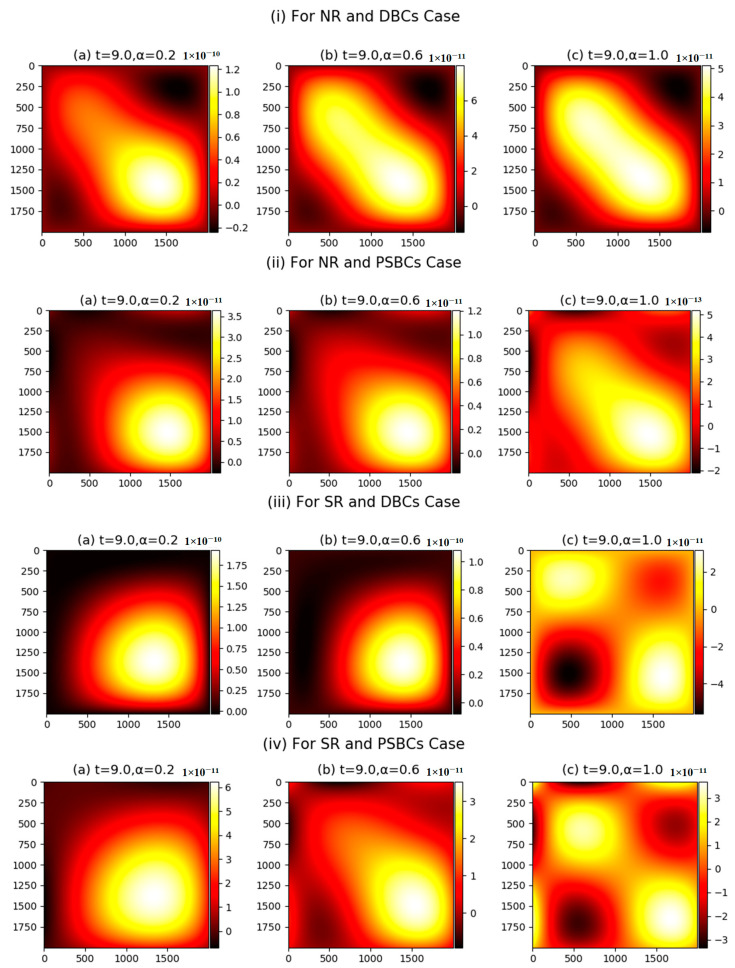
(**i**–**iv**) Density plots with $M_{1}=M_{2}=M_{3}=M_{4}=5,N=1000$.

In order to study the numerical impact of the defined parameters, we have offered here higher-dimensional density plots at t=9.0 in Figure 7. There are four different cases, we can see in the presented Figure 7.

When the initial data are applied, a considerable disturbance is observed. Thus, the energy (or momentum) transformation process began abruptly as a result of the massive burst of initial data. Additionally, the Dirichlet boundary conditions are included to demonstrate that boundaries have no flux. At specific time intervals, due to their identical energy levels, some plasma particles form clusters, which are articulated behind a thin layer (see Figure 7i(a)). As a result, the transformation of plasma particles from high density to low density occurs naturally until the equilibrium condition is reached.

The coated layer expands itself due to the transformation of plasma particles, which can be seen at t=9.0,α=0.6 (see Figure 7i(b)). The dark portion of the domain shows that there are no excited plasma particles is found in this area, and it is reducing gradually because plasma particles inside the coated layer are trying to adjust itself. To acquire the equilibrium condition, movement of immense volume of plasma particles can be perceived from the upper part to the lower part with integer α (see Figure 7i(c)). Further in Figure 7ii, we can see the variations produced due to the partial slip boundary conditions. At t=9.0,α=0.2, particles formulate the cluster of plasma particles, which can be seen (see Figure 7ii(a)) between the mesh domains 1000≤x,y≤2000.

Some quantity of volume of excited plasma particles (PP) can also be seen at the other position of the domain, especially at the boundaries of the computational domain, which is due to the slip effects. Two types of movements can be seen in further Figure 7ii(b,c), i.e., the transformation of plasma particles inside the layer and at the boundaries of the domain. In the final shape, i.e., at t=9.0,α=1.0, the high density of excited plasma particles is placed at the boundaries. Figure 7iii,iv are strategized for different values of the fractional and integer values of the α. The SR parameter is provided to boost up the plasma particles on a large scale. Therefore, we can see the procedure to formulate the small bunches in Figure 7iii,iv.

**Figure 8:**
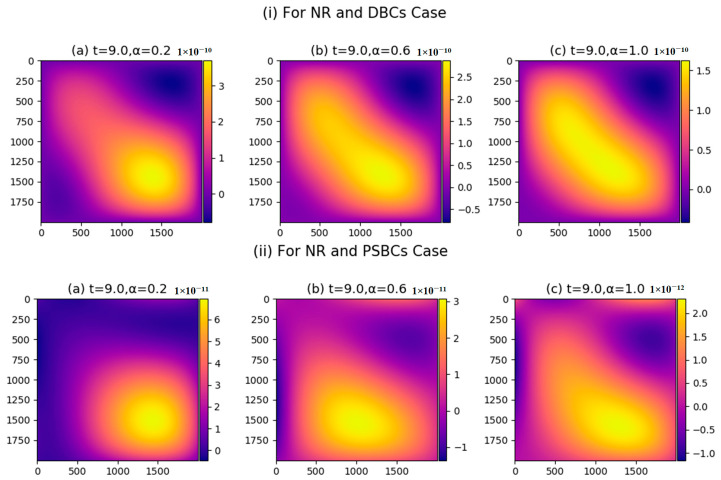

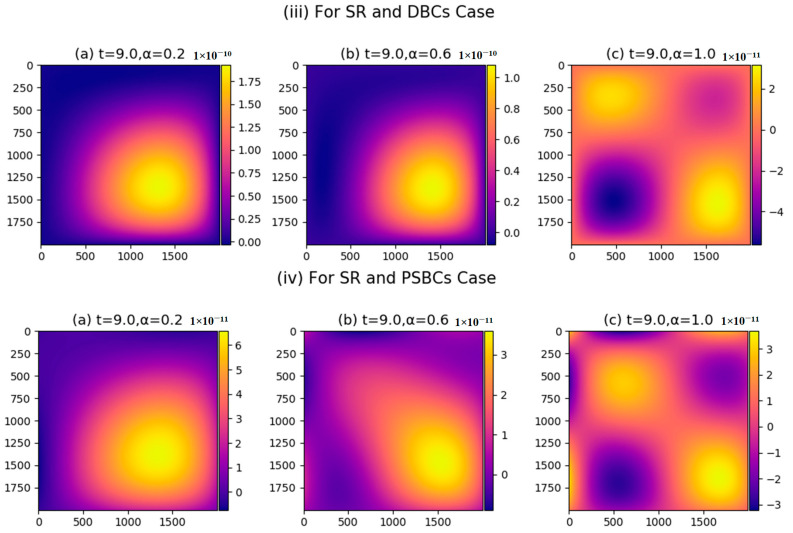
(**i**–**iv**) Current plot $M_{1}=M_{2}=M_{3}=M_{4}=5,N=1000$.

Parametric study of current is explained in Figure 8, which shows that current density has high impact between 1000≤x,y≤2000 (see Figure 8i(a)–iv(a). The reason behind this happening is that huge quantity of ions and electrons are accessible in the discussed domain. It is also witnessed that the current pattern is quite similar to the density profile. It is obvious because that current density is high, where high energy particles movement is recorded. Similarly, we can perceive the other parametric attitude of the current in Figure 8.

## 4. Concluding Remarks

We articulated an upgraded version of the Vlasov Maxwell system and then used a semi-spectral numerical method to define the numerical solution with partial slip boundaries. Additionally, a detailed discussion of the main aims and an analysis of the results are presented. The following are some closing points:

- Due to the SR parameter, plasma particles can withstand a high rate of destruction. As a result, the density performance of the SR case is completely different.
- Plasma particles scatter to different positions and are further arranged in a cluster form. With increasing values of α, this cluster expands itself under the coated layer.
- The fractional parameter established a new tradition for studying this subject in novel ways. It enables us to investigate the concealed figures of plasma particles.
- The PSBCs parameter assists the plasma particles to obtain more energy from the boundaries and further disperses it to the different positions of the computational domain.
- Although the specified problem contains numerous complications, the technique effectively handles them and produces extremely accurate and stable results that are demonstrated using dissimilar methodology.
- As described previously, this approach is expanded to higher dimensions in this article. As a result, we may conclude that the technique is also efficient, well-matched, and compatible with higher-dimensional problems.

## Data Availability

Not applicable.
